# Safety of prostaglandins in the peripartum period in case of sickle‐cell disease: A multicenter cohort study

**DOI:** 10.1002/pmf2.70014

**Published:** 2025-08-21

**Authors:** Mayi Gnofam, Jeanne Sibiude, Louis Affo, Frédéric Galacteros, Edouard Lecarpentier, Florence Wang, Marine Driessen, Olivier Graesslin, Gylna Loko, Giovanna Cannas, Stéphane Bounan, Hervé Fernandez, Véronique Debarge, Caroline Makowski, Laurent Mandelbrot, for the Drepagest Study Group

**Affiliations:** ^1^ Department of Obstetrics and Gynecology Assistance Publique‐Hôpitaux de Paris Hôpital Louis Mourier, and FHU PREMA Colombes France; ^2^ Université Paris Cité Paris France; ^3^ Inserm IAME Paris France; ^4^ Department of Internal Medicine Assistance Publique‐Hôpitaux de Paris Hôpital Louis Mourier Colombes France; ^5^ Department of Internal Medicine Assistance Publique‐Hôpitaux de Paris Red Cell Genetic Disease Unit GHU Henri Mondor U‐PEC, Paris, France; ^6^ Department of Obstetrics and Gynecology Centre Hospitalier Intercommunal Créteil Université Paris‐Est Créteil, IMRB INSERM U955 Créteil France; ^7^ Department of Obstetrics and Gynecology Assistance Publique‐Hôpitaux de Paris Hôpital Necker AP‐HP Paris France; ^8^ Department of Obstetrics and Gynecology Hôpital Maison Blanche Reims France; ^9^ Sickle Cell Disease Reference Center Hôpital La Meynard Martinique Fort‐de‐France France; ^10^ Sickle Cell Disease Reference Center Hôpital E. Herriot HCL Lyon France; ^11^ Department of Obstetrics and Gynecology Hôpital Delafontaine Saint Denis France; ^12^ Department of Obstetrics and Gynecology Assistance Publique‐Hôpitaux de Paris Hôpital Bicêtre Le Kremlin Bicêtre France; ^13^ Department of Obstetrics and Gynecology Centre Hospitalier Universitaire de Lille Lille France; ^14^ Department of Internal Medicine Centre Hospitalier Universitaire Grenoble Alpes Grenoble France

**Keywords:** dinoprostone, labor induction, misoprostol, postpartum hemorrhage, prostaglandins, sickle‐cell disease, sulprostone

## Abstract

**Introduction:**

Pregnancies with sickle‐cell disease are at high risk of maternal, obstetrical, and fetal complications, for which prostaglandins are often indicated to induce labor or to treat postpartum hemorrhage. There is concern that prostaglandin use may increase the risk of vaso‐occlusion, but to date no clinical study has yet assessed this risk. Our objective was to assess the safety of prostaglandins during delivery and postpartum in women with sickle‐cell disease.

**Method:**

A retrospective multicenter cohort study in 10 tertiary university‐affiliated centers in France, including all women with sickle‐cell disease (S/S, S/C, or S/β‐thalassemia) delivering in the centers.

**Methods:**

We compared pregnancies with and without exposure to prostaglandins, overall and specifically according to whether prostaglandins were used for labor induction or for postpartum hemorrhage. The main outcome was the occurrence of vaso‐occlusive crisis and/or thromboembolism, within 7 days after delivery. Secondary outcomes were vaso‐occlusive crises requiring intravenous morphine and/or intensive care admission.

**Results:**

Prostaglandins were used in 114/411 pregnancies (27.7%). In 106/411 (25.8%), they were used to induce labor, with Prostaglandin E2 (dinoprostone gel or pessary) for 95 patients and misoprostol for 11 patients. The incidence of postpartum hemorrhage was 60/411 (14.6%), and 11 cases required second‐line therapy with prostaglandins; sulprostone was used as per French practice guidelines. The incidence of vaso‐occlusive crises and/or venous thromboembolism in the postpartum did not differ between patients exposed and not exposed to prostaglandins (14/114 (12.2%) vs. 34/293 (11.6%), respectively; *p* = 0.87), nor did the incidence of severe vaso‐occlusive crises requiring morphine and/or intensive care unit hospitalization (7.0% vs. 7.8%, respectively; *p* = 0.78). Among patients undergoing labor induction, acute complications occurred in 12/106 (11.6%) exposed to prostaglandins versus 6/77 (7.6%) unexposed (*p* = 0.46). Among patients with postpartum hemorrhage, rates were respectively 2/11 (18.2%) versus 9/49 (18.4%; *p* = 0.99).

**Conclusion:**

The use of prostaglandins in patients with sickle‐cell disease for labor induction or for postpartum hemorrhage appeared safe, within the limitations of a retrospective study.

## 1 INTRODUCTION

Sickle‐cell disease, which is due to a mutation in the beta‐globin gene, is the most frequent autosomal recessive disease in the world [1]. Homozygous hemoglobin S as well as compound heterozygous variants (S/C, S/β‐thalassemia, S/D Punjab, or S/ O‐Arab hemoglobin) result in hemolytic anemia. Complications include vaso‐occlusive crises; acute chest syndrome; venous or arterial thromboembolisms; and chronic kidney, heart, and pulmonary diseases and infections [2]. Pregnancy is an important risk factor for acute complications, maternal and fetal morbidity–mortality [3]. Thus, pregnancies with sickle‐cell disease require close monitoring, preferably in reference centers [4, 5, 6].

Induction of labor is considered part of the management of pregnancies with sickle‐cell disease, particularly in the case of complications. Prostaglandins, including analogs of prostaglandin E2, F2a, or E1 [7] are commonly used to induce labor. Prostaglandins are also used for postpartum hemorrhage [8]. However, there is concern that prostaglandins may increase the risk of vaso‐occlusive crisis. This fear is based on historical case studies [9, 10] and some experimental data which showed sickling of red cells from sickle‐cell disease patients when exposed to prostaglandin F2 [11]. In contrast, an in vitro study failed to show an increase in sickling when HbSS‐laden erythrocytes were exposed to PGE2 [12].

Prostaglandins E2 and E1 used in obstetrics both induce vasodilatation, but also have a pro‐inflammatory action, which could induce stress and thus increase the risk of a vaso‐occlusive crisis [13, 14] Patients with sickle‐cell disease have higher basal rates of prostaglandin E than the general population, suggesting a proinflammatory state, and these levels are reported to increase significantly during pregnancy [15, 16]. Sickle‐cell disease is a chronic inflammatory condition with elevated leucocyte counts and cytokine levels, notably interleukin‐10, macrophage inflammatory protein 1α, soluble CD40 ligand and placenta growth factor. Prostacyclin is decreased and prostaglandin E2 is increased in the steady state, and PGE2 is further elevated during vaso‐occlusive events [13]. The release of proinflammatory mediators including prostaglandin E2 may contribute to the onset of acute chest syndrome [14]. Additionally, prostaglandins, as well as leukotrienes, sensitize the nociceptor, which can lead to increased pain. In a mouse model with sickle‐cell disease increased levels of prostaglandin E caused sensitization of the nociceptors with hyperalgesia, which was reversed by blocking its synthesis [17].

To our knowledge, the risk of vaso‐occlusive crisis associated with prostaglandins has not yet been evaluated in sickle‐cell disease pregnancies. Our objective was to assess the safety of prostaglandins during delivery and postpartum in women with sickle‐cell disease.

## 2 METHODS

We conducted a retrospective exposed/unexposed study in 10 French centers with a maternity and a sickle‐cell disease competence or reference center. All pregnancies with sickle‐cell disease (S/S, S/C, or S/β‐thalassemia) identified in the centers’ databases with deliveries after 23 weeks of gestation, from January 1, 2009 to June 30, 2020 were included, except for patients who expressed opposition to the use of their medical data for research. For patients who had several deliveries during that timeframe, in order not to include the same patient more than once in the analysis, only the last delivery was considered in the analyses. The database was used in a previous study concerning the risk of corticosteroids in sickle‐cell disease pregnancies [18].

Three analyses were conducted. The first compared the population exposed to any prostaglandin (PG) to the unexposed. The second analysis compared patients who had labor induction exposed versus unexposed to prostaglandins. The third analysis compared patients with postpartum hemorrhage treated with sulprostone versus no prostaglandins.

Primary outcome was the occurrence of an acute complication, defined as a vaso‐occlusive crisis and/or venous thromboembolism, within 7 days after delivery. Secondary outcomes were vaso‐occlusive crises requiring intravenous morphine and/or intensive care admission. We also compared the incidence of blood transfusion within 7 days after delivery, severe postpartum hemorrhage, cesarean section for failed labor induction and perinatal death.

For induction of labor, the prostaglandins used are PGE2 analog dinoprostone (gel or pessary) and PGE1 analog misoprostol (tablets administered orally or vaginally). Other methods of induction include balloon ripening, oxytocin, and artificial rupture of the membranes. French guidelines contra‐indicate the use of prostaglandins for induction in the case of a scarred uterus. Protocols differed between centers and methods could be combined.

For postpartum hemorrhage, French guidelines recommend intravenous sulprostone, a PGE2 analog (500 µg in 30 min followed by 500 µg in 4 h if necessary) when manual exploration of the uterus, intravenous oxytocin (maximum 20 UI), inspection of the lower genital tract in case of vaginal delivery, and administration of tranexamic acid are insufficient [8]. If sulprostone is ineffective, the next steps are uterine balloon tamponade, embolization, and/or surgery.

Besides maternal, pregnancy, delivery, and neonatal characteristics, we collected variables concerning sickle‐cell disease, including the type (S/S/, S/C, or S/β‐thalassemia) and severity of the disease, and management during pregnancy. Severe baseline disease was defined by one or more of the following: a history of hospitalization in an intensive care unit for vaso‐occlusive crisis or acute chest syndrome, more than three crises in a year requiring hospitalization, frequent crises with a major impact on the quality of life according to the medical reports of the referring internist physician, or organ‐related complications.

Data were collected in each center from medical records, and anonymized and managed using a secure REDCap electronic database [19].

We performed the statistical analysis using IBM SPSS Statistics v27. Categorical variables were analyzed with the Chi Square or Fisher test as appropriate. Continuous variables were analyzed using the Student test. The cut‐off for significance used was a *p* value < 0.05.

The study was approved by the Institutional Review Board (Comité d'Evaluation de la Recherche Paris Nord N°CER‐2020‐63, IRB 00006477) on January 22, 2021. Patients were informed at the time of hospitalization that data from their medical records could be used for research purposes unless they refused.

## 3 RESULTS

Among 497 pregnancies with sickle‐cell disease, we included 411 deliveries and excluded 5 with deliveries before 23 weeks gestation, 32 for whom data were not available and 49 previous pregnancies in the case of repeat pregnancies in the same patients. The type of sickle‐cell anemia was mostly S/S (61.1%), with 27.0% S/C and 11.7% S/ß‐thalassemia. Over half had a severe disease. The majority of patients (77.4%) had a history of transfusion and 13.1% had posttransfusion complications, mostly alloimmunization, delayed hemolytic reaction, or iron overload. During pregnancy, 228 patients (55.5%) had at least one vaso‐occlusive crisis, 39.4% (*n* = 162) required morphine, 60.7% (*n* = 247) received transfusions, and 11.9% (*n* = 49) required hospitalization in intensive care. There was no maternal death. Fetal demise occurred in 12 cases (2.9%) and there was one neonatal death (0.2%).

### 3.1 Comparison between patients exposed and not exposed to prostaglandins (Tables 1 and 2)

Patients exposed to prostaglandins, in comparison with the unexposed (Table 1), were younger (28.5 years vs. 30.8 years, *p* < 0.001), more nulliparous (68.4% vs. 36.3%, *p* < 0.001), less likely to have a previous cesarean (4.4% vs. 37.5%, *p* < 0.001), less likely to have a thromboembolic event before or during the pregnancy (respectively 7.0% vs. 15.0%, *p* = 0.03 and 0% vs. 3.8%, *p* = 0.04), received less low‐dose aspirin during pregnancy (3.5% vs. 11.6%, *p* = 0.01), and had less preeclampsia (8.8% vs. 17.1%, *p* = 0.04) (Table 1). There was no significant difference between the exposed and unexposed regarding the proportion of S/S type or history of severe disease.

**TABLE 1:** Baseline characteristics of patients with sickle‐cell disease between pregnancies exposed and not exposed to prostaglandins.

	Exposed (*n* = 114)		Not exposed (*n* = 293)		
	*n* (%)		*n* (%)		*p*
Age (mean years, SD)	28.5	5.3	30.8	5.4	< 0.001
Geographical origin of family					
Sub‐Saharan Africa	86	75.4	223	76.1	0.89
Antilles	22	19.2	44	15.0	0.30
Other	3	2.6	23	7.8	0.07
BMI (mean kg/m^2^, SD)	22.9	4.3	23.5	5.0	0.31
Active smoking	5	4.4	4	1.4	0.12
Parity (mean, SD)	0.4	0.6	1.1	1.2	< 0.001
Twin pregnancies	9	7.9	18	6.1	0.99
History of venous thromboembolism	8	7.0	44	15.0	0.03
Previous cesarean section	5	4.4	110	37.5	< 0.001
History of postpartum hemorrhage	1	0.9	9	3.1	0.99
History of blood transfusion	85	74.6	233	79.5	0.36
Severe disease^a^	54	47.4	153	52.2	0.57
Type of SCD					0.48
S/S	74	64.9	173	59.0	
S/C	24	21.1	87	29.7	
S/β‐thalassemia	16	14.0	32	10.9	
Baseline hemoglobin concentration before conception (mean g/dL, SD)	8.9	1.2	9.2	1.4	0.18
Therapy during pregnancy					
Hydroxyurea/hydroxycarbamide	2	1.8	8	2.7	0.73
Low‐dose aspirin	4	3.5	34	11.6	0.01
Low molecular weight heparin	11	9.6	51	17.4	0.06
Oxygen therapy					0.39
For vaso‐occlusive crises	36	31.6	110	37.5	
Prophylactic	18	15.8	48	16.4	
None	59	51.8	129	44.0	

Abbreviations: BMI, body mass index; PCA, patient controlled analgesia; SD, standard deviation.

^a^A severe form of the disease defined by one or more of the following: a history of hospitalization in an intensive care unit for vaso‐occlusive crisis, a history of acute chest syndrome, more than three crises in a year requiring hospitalization, frequent crises with a profound impact on the quality of life according to the medical reports of the referring internist physician, or visceral complication.

Regarding the primary outcome (occurrence of vaso‐occlusive crisis and/or venous thromboembolism within 7 days after delivery), no significant difference was found between the groups of patients exposed and unexposed to prostaglandins (*n* = 14, 12.2 % vs. *n* = 34, 11.6% *p* = 0.87) (Table 2). Considering secondary outcomes, there was no difference in the incidence of severe crises requiring intensive care admission or intravenous morphine use (Table 2). Obstetrical outcomes differed between the two groups, with more vaginal deliveries in patients exposed to prostaglandins (*n* = 64, 56.1% vs. *n* = 92, 31.4%, *p* < 0.001) and in the case of cesarean section, the indication was more frequently failed induction (*n* = 23, 20.1% vs. *n* = 16, 5.4%, *p* < 0.001). Mean gestational age at delivery was higher (37.7 weeks vs. 36.7 weeks, *p* = 0.007).

**TABLE 2:** Comparison of pregnancy, delivery, and postpartum outcomes between patients exposed and not exposed to prostaglandins (*n* = number of patients, each patient could have more than one complication).

	Exposed		Not exposed		
	(*n* = 114)		(*n* = 293)		
	*n*	%	*n*	%	*p*
Complications during the pregnancy					
Threatened preterm labor	3	2.6	15	5.1	0.42
Preeclampsia	10	8.8	50	17.1	0.04
Fetal growth restriction	12	10.5	38	13.0	0.72
Intrauterine fetal demise	5	4.4	4	1.4	0.12
Pneumonia	9	7.9	10	3.4	0.07
Blood transfusion	70	61.4	177	60.4	0.73
Number of emergency transfusions (mean, SD)	1.4	0.6	1.3	0.8	0.61
VOC during pregnancy, postpartum excluded	55	48.2	173	59.0	0.07
Number of VOC (mean, SD)	1.8	1.1	2.0	1.4	0.39
Use of morphine during pregnancy	39	34.2	123	42.0	0.85
Acute thoracic syndrome (ATS)	8	7.0	31	10.6	0.68
Thromboembolic event during pregnancy	0	0.0	11	3.8	0.04
Characteristics of labor and delivery					
Gestational age (mean, SD)	37.7	3.2	36.7	3.3	0.007
Hemoglobin at delivery g/dL (mean, SD)	9.0	1.5	8.9	1.6	0.38
Prolonged labor	20	17.5	20	6.8	0.002
Fetal heart rate abnormalities	39	34.2	81	27.6	0.15
Fever during labor (≥38°C)	11	9.6	11	3.8	0.03
Type of anesthesia					
General anesthesia	7	6.1	23	7.8	0.68
No anesthesia	6	5.2	4	1.4	0.03
Intravenous morphine	3	2.6	6	2.0	0.72
Epidural analgesia	90	67.1	134	45.7	<0.001
Spinal anesthesia	11	9.6	149	50.9	<0.001
Mode of delivery					
Vaginal delivery	64	56.1	92	31.4	<0.001
Cesarean section	47	41.2	201	68.6	<0.001
Cesarean section for failed induction	23	20.1	16	5.4	<0.001
Postpartum outcomes, within 7 days after delivery					
Any VOC and/or thromboembolism, composite primary outcome	14	12.2	34	11.6	0.87
VOC	12	10.5	30	10.2	0.96
Severe VOC^a^	8	7.0	23	7.8	0.78
ATS after delivery	5	4.4	12	4.1	0.84
Morphine for VOC	9	7.9	24	8.2	0.35
Venous thromboembolism	5	4.4	9	3.1	0.55
Blood transfusion	26	22.8	48	16.4	0.15
Severe PPH	15	13.1	11	3.7	<0.001
Intensive care unit (ICU) hospitalization during pregnancy or postpartum	14	12.4	38	13.1	0.85
ICU hospitalization for VOC or ATS	5	4.4	25	8.5	
ICU hospitalization for other reasons	9	8.0	13	4.6	

Abbreviations: ATS, acute thoracic syndrome; PPH, postpartum hemorrhage; SD, standard deviation; VOC, vaso‐occlusive crisis.

^a^ Severe VOC is defined by admission to an intensive care unit, acute chest syndrome, or use of morphine.

### 3.2 Comparison between patients exposed and not exposed to prostaglandins among patients undergoing induction of labor

Of 183 patients (44.5% of the cohort) who had labor induction, the indications were sickle‐cell disease in the majority (*n* = 119, 65%), fetal conditions including non‐stress test abnormalities (*n* = 21, 25.3%), fetal growth restriction (*n* = 7, 3.8%), hypertension (*n* = 15, 8.2%), other maternal conditions, including gestational diabetes and cholestasis (*n* = 14, 7.7%), and intrauterine infection (*n* = 4, 2.2%).

Various methods of induction were used individually or in combination, as per local protocols, including prostaglandins, oxytocin, cervical ripening balloon, and artificial rupture of membranes. One or more prostaglandins were used in 106/183 (57.9%) labor inductions, 39 (36.8%) with dinoprostone gel (1mg or 2mg), 66 (62.2%) with a dinoprostone pessary (10 mg), and 11 (10.4%) with misoprostol. Inductions without prostaglandins included 41 with oxytocin (53.2%), 42 with balloon ripening (54.5%), and 31 with artificial membrane rupture (40.3%). Exposed, versus unexposed, patients were significantly younger (28.1 years (SD: 5.3) vs. 30.4 years (SD: 5.1), *p* = 0.004), had fewer pregnancies (parity: 0.3 vs. 1.1 *p* < 0.001), less low‐dose aspirin (2.8% vs. 13.5%, *p* = 0.02), and fewer pulmonary infections during pregnancy (1.3% vs. 8.4% *p* = 0.039). They did not differ for other characteristics especially sickle‐cell disease severity or complications during pregnancy (Table 3).

**TABLE 3:** Characteristics of patients exposed and not exposed to prostaglandins among women undergoing induction of labor.

	Exposed *n* = 106		Not exposed *n* = 77		
	***n***	**%**	***n***	**%**	*p*
Age (mean, SD)	28.1	5.3	30.4	5.1	0.004
Geographic origin of family					
Sub‐Saharan Africa	79	74.5	60	77.9	0.73
Antilles	22	20.8	14	18.1	0.71
Other	2	1.9	4	5.1	0.24
BMI (mean, SD)	22.9	4.2	23.2	5.1	0.63
Active smoking	5	4.7	2	2.6	0.70
Parity (mean, SD)	0.3	0.6	1.1	1.2	<0.001
History of venous thromboembolism	7	6.6	10	13.0	0.20
Comorbidities^a^	25	23.6	19	24.7	0.86
Previous uterine scar	3	2.8	13	16.9	0.09
Severe sickle‐cell disease^b^	51	48.1	31	40.2	0.29
Baseline hemoglobin level before pregnancy (mean, SD)	8.9	1.2	9.3	1.4	0.06
Transfusion during the pregnancy	65	61.3	40	51.9	0.17
Vaso‐occlusive crisis during pregnancy	54	50.9	45	58.4	0.22
Thromboembolism during pregnancy	0	0.0	2	2.6	0.17
Antenatal corticosteroids	2	1.9	6	7.8	0.32
Gestational age, WG (mean, SD)	37.7	3.4	38.0	1.6	0.39
Indications for induction of labor					
Sickle‐cell disease	87	82.1	62	80.5	0.58
FGR	7	6.6	1	1.3	0.10
Intrauterine infection	2	1.9	2	2.6	0.71
Other fetal conditions	14	13.2	10	13.0	0.92
Hypertension	9	8.5	8	10.4	0.58
Other maternal conditions	10	9.4	6	7.8	0.79

Abbreviations: BMI, body mass index; FGR, fetal growth restriction; WG, weeks' gestation; SD, standard deviation.

^a^ Comorbidities defined by the presence of other medical conditions not related to sickle‐cell disease

^b^ Severe form of the disease defined by one or more of the following: a history of hospitalization in an intensive care unit for vaso‐occlusive crisis, a history of acute chest syndrome, more than three crises in a year requiring hospitalization, frequent crises with a profound impact on the quality of life according to the medical reports of the referring internist physician, or visceral complication.

No difference was found between patients exposed and not exposed to prostaglandins for the primary outcome (*n* = 12 (11.6%) vs. *n* = 6 (7.2%), *p* = 0.46) nor for secondary outcomes or obstetrical outcomes (Table 4).

**TABLE 4:** Comparison of intrapartum and postpartum outcomes between patients exposed and not exposed to prostaglandins for induction of labor (*n* = number of patients, each patient could have more than one complication).

	Exposed *n* = 106		Not exposed *n* = 77		
	n	%	*n*	%	*p*
Sickle‐cell disease outcomes					
Any VOC and/or thromboembolism, composite primary outcome	12	11.6	6	7.2	0.46
VOC after delivery	11	10.7	6	7.2	0.61
Severe VOC after delivery^a^	8	7.4	3	3.6	0.36
ATS after delivery	5	4.1	0	0	0.09
Thromboembolism after delivery	4	3.3	0	0	0.14
Use of morphine for VOC after delivery	9	8.3	4	4.8	0.58
Blood transfusion after delivery	21	19.8	8	10.8	0.10
Transfer to intensive care unit for VOC or ATS	5	4.7	2	2.6	0.70
Obstetrical outcomes					
Cesarean section for failure to progress or failed induction of labor	23	21.5	14	19.3	0.58
Cesarean section for VOC	2	1.7	0	0	0.51
Cesarean section for fetal indication	9	9.1	7	9.6	0.89

Abbreviations: ATS, acute thoracic syndrome; VOC, vaso‐occlusive crisis.

^a^ Severe VOC is defined by admission to an intensive care unit or use of morphine.

### 3.3 Comparison between patients exposed and not exposed to prostaglandins for postpartum hemorrhage

The incidence of postpartum hemorrhage was 14.6% (*n* = 60), and severe PPH was 5.8% (*n* = 24). Among the 60 patients with PPH, 11 (18.3%) received sulprostone. The mean dose of sulprostone was 625 µg (SD: 231.46). Surgical procedures were required in 13 cases (21.7% of the PPH) including 3 hysterectomies. Patients who received sulprostone had a lower proportion of preeclampsia (7.1% vs. 28.1%, *p* = 0.037), but did not differ in other characteristics (Table 5).

**TABLE 5:** Characteristics of patients exposed and not exposed to prostaglandins among women with postpartum hemorrhage.

	Exposed *n* = 11		Not exposed *n* = 49		
	*n*	%	*n*	%	*p*
Age (mean, SD)	31.3	4.3	29.9	4.9	0.38
Origin					
Sub‐Saharan Africa	10	90.9	41	83.7	0.54
Antilles	0	0.0	3	6.1	0.40
Others	1	9.0	4	8.2	0.92
BMI (mean, SD)	23	5.2	23.6	5.1	0.72
Active smoking	1	9.0	2	4.1	0.47
Parity (mean, SD)	0.8	0.6	0.5	0.9	0.38
History of any thromboembolism	1	9.1	7	14.3	0.72
Comorbidities	3	27.2	18	36.7	0.85
History of PPH	1	9.0	1	2.0	0.48
Severe form of the disease	5	45.5	33	67.3	0.70
Baseline hemoglobin level (mean, SD)	8.5	1.2	8.7	1.5	0.84
Transfusion during the pregnancy	8	72.7	30	61.2	0.73
VOC during the pregnancy	5	45.5	29	59.1	0.79
Thromboembolism during pregnancy	0	0.0	2	4.1	0.51
Gestational age in weeks (mean, SD)	37.6	1.0	36.7	3.7	0.39
Hemoglobin at delivery (mean, SD)	8.3	1.6	8.8	1.6	0.47
Prolonged labor	2	18.2	6	12.2	0.64
Mode of delivery					0.26
Vaginal delivery	4	36.4	10	20.4	
Cesarean section	7	63.6	39	79.6	
Total blood loss in milliliters (mean, SD)	1442.0	878.4	927.7	479.1	0.01
Oxytocin administered after delivery	10	90.9	38	77.6	0.67
Carbetocin administered after delivery	1	9.1	5	10.2	0.86
Tranexamic acid after delivery	3	27.2	8	16.3	0.41

Abbreviations: BMI, body mass index; PPH, postpartum hemorrhage; SD, standard deviation; VOC, vaso‐occlusive crisis.

No significant difference was found between patients exposed and not exposed to sulprostone in terms of the primary outcome (*n* = 2, 18.2% vs. *n* = 9, 18.4%, *p* = 0.99). Women exposed to sulprostone had more blood transfusions after delivery, as expected in the context of postpartum hemorrhage.

No difference was shown between the groups of patients exposed and not exposed to sulprostone in terms of other secondary outcomes (Table 6).

**TABLE 6:** Comparison of adverse outcomes between patients exposed and not exposed to prostaglandins for postpartum hemorrhage (*n* = number of patients, each patient could have more than one complication).

	Exposed *n* = 11		Not exposed *n* = 49		
	*n*	%	*n*	%	*p*
Sickle‐cell disease outcomes :					
Any VOC and/or thromboembolism, composite primary outcome	2	18.2	9	18.4	0.99
Severe VOC after delivery^a^	0	0	8	16.3	0.34
ATS after delivery	0	0	7	14.3	0.05
Intensive care unit hospitalization for VOC or ATS	0	0	7	14.3	0.33
Thromboembolism after delivery	1	9.0	6	12.2	0.75
Obstetrical outcomes:					
Severe PPH	7	63.6	17	34.7	0.08
Transfer to intensive care unit for PPH	3	27.2	6	12.2	0.35
Blood transfusion after delivery	9	81.8	23	46.9	0.04
Surgical treatment for PPH	6	54.5	5	10.2	0.56

Abbreviations: ATS, acute thoracic syndrome; PPH, postpartum hemorrhage; VOC, vaso‐occlusive crisis.

^a^ Severe VOC is defined by admission to an intensive care unit, acute chest syndrome, or use of morphine.

## 4 DISCUSSION

### 4.1 Main findings

Prostaglandin use was not associated with an increased risk of severe complications of sickle‐cell disease. These results were consistent in the subgroups treated for induction of labor and postpartum hemorrhage. We found no difference in the type or severity of sickle‐cell disease between patients exposed or not exposed to prostaglandins which could have biased the results. However, regarding induction of labor, our reassuring results concerned mainly prostaglandin E2 and more data is necessary for misoprostol.

It should be stressed that while the main outcome measured, vaso‐occlusive crises or thromboembolism in the postpartum occurred in 12% of patients, the proportion of patients who had one or more vaso‐occlusive crises during the pregnancy was much higher at 56%. Adverse maternal and fetal events were also frequent, including preeclampsia, small for gestational age and fetal demise. The rate of cesarean section was over one half. These outcomes were expected since pregnancy is associated with an increase in complications of sickle‐cell disease, and sickle‐cell disease is associated with poorer pregnancy outcomes including maternal morbidity, a high cesarean section rate and perinatal morbidity when compared to the general population [3, 20]. These outcomes are a challenge for clinical practice [21, 22, 23].

### 4.2 Strengths and limitations

This study is the first to our knowledge to investigate the impact of prostaglandins in pregnant women with sickle‐cell disease. The study population was large. Another strength is the multicenter design, with a diversity of protocols in different centers, some centers prohibiting and others authorizing prostaglandins in women with sickle‐cell disease. This reduces prescription bias and is a factor of external validity. In fact, adverse obstetrical and hematologic outcomes were consistent with those previously reported in sickle‐cell disease pregnancies [3, 24]. Moreover, data collection was exhaustive and missing data were rare. Finally, since the two populations of patients exposed and not exposed were very comparable both in the principal analysis and in the analyses on restricted populations, we believe that selection bias is unlikely to influence our main finding on the safety of prostaglandin use.

The main limitation of our study is its retrospective nature. Since the study spans a period of over 10 years, changes in the management of sickle‐cell disease and especially labor induction have occurred over time. Also, few centers systematically measured HbS and HbA levels at the time of delivery. Bishop scores were not collected, but they were unlikely to be related to sickle‐cell disease complications. However, despite these missing data, information was available in all medical records to analyze the primary outcome and the exposure variables. Postpartum outcomes could not be studied reliably more than 7 days after delivery because longer‐term follow‐up could be done in other institutions. The population was insufficient to permit an analysis stratified on the type of prostaglandin, and few patients received misoprostol. Finally, the definition of the severity of disease can be challenged, since there is no validated standard definition in the literature. Other criteria may have been considered for the definition of severity, such as chronic treatment or frequency of blood transfusion outside of pregnancy. However, the two groups remained comparable after considering any variable that could be linked to severity of disease. Thus, it is very unlikely that any change in the definition would have changed the results of the statistical analysis.

### 4.3 Interpretation

We searched MEDLINE on PubMed with the keywords “sickle cell” AND “prostaglandin” AND “pregnancy” OR “abortion” (search conducted on May 9, 2021 and updated on February 22, 2025) and retrieved only two case reports of the use of prostaglandins in pregnant sickle‐cell disease patients. One is a case report of vaso‐occlusive crisis in a patient receiving prostaglandin for induction of labor at 38 weeks [10]. The other case was a fat embolism occurring a few hours after a second‐trimester termination of pregnancy in a triplet pregnancy induced with intravenous prostaglandin E2, which resulted in maternal death [25]. There are few data on small populations regarding the tolerance of prostaglandins in sickle‐cell disease outside pregnancy.

### 4.4 Clinical and research implications

Management of induction of labor and postpartum hemorrhage is crucial for patients with sickle‐cell disease and prostaglandins are useful in both situations. Many guidelines recommend scheduling delivery between 38 and 40 weeks gestation in sickle‐cell disease patients, because the risk of sickle‐cell crises and of perinatal morbidity and mortality (placental abruption, stillbirth, pre‐eclampsia, peripartum cardiomyopathy) increases in late pregnancy [20, 21].

In the general French obstetrical population [26], 26% had induction of labor, with prostaglandins in nearly two‐thirds. In our cohort, the proportion of induction was 45% and 48% of inductions used prostaglandins, suggesting that practice in sickle‐cell disease patients is to induce more but to restrict prostaglandin use.

For postpartum hemorrhage, our findings indicate that sulprostone can be used in sickle‐cell disease patients. This is of crucial importance since blood loss leading to hypovolemia increases the risk of vaso‐occlusive crisis [27]. The incidence of postpartum hemorrhage (14.7%) appeared slightly higher in our sickle‐cell disease population than in the general obstetrical population in France, where it was 11.6%, while severe postpartum hemorrhage had a higher incidence than in the general population (5.8% versus 1.8%).

Further large‐scale prospective multicentric studies are necessary to explore the impact of specific prostaglandin drugs, particularly misoprostol.

## 5 CONCLUSION

Our study suggests that prostaglandin use is safe in pregnant women with sickle‐cell disease.
